# Supplementary material for: Development of an antigen detection test kit (Melioidosis-ATK) for point-of-care diagnosis of melioidosis
Source: Microbiol Spectr. 2026 Mar 27;14(5):e02881-25. doi: 10.1128/spectrum.02881-25 (PMC13142003; doi:10.1128/spectrum.02881-25)
Supplement: Table S1 — Non-B. pseudomallei pathogens identified by culture from various clinical specimens (N = 204) and their results when tested with the Melioidosis-ATK. [file spectrum.02881-25-s0001.pdf]

Table S1: Non-*B. pseudomallei* pathogens identified by culture from various clinical specimens (N=204) and their results when tested with the Melioidosis-ATK.

Sample Type (Total no.)	Organism Group	Species	No. of Samples	Negative by Melioidosis-ATK, n (%)
Blood culture (46)	Gram-negative bacteria	*Acinetobacter baumannii*	2	2 (100)
		Enterobacter cloacae	1	1 (100)
		Escherichia coli	4	4 (100)
		Klebsiella pneumoniae	3	3 (100)
		Roseomonas mucosa	1	1 (100)
		Salmonella sp.	1	1 (100)
	Gram-positive bacteria	*Bacillus* sp.	3	3 (100)
		Corynebacterium afermentans	1	1 (100)
		Corynebacterium sp.	3	3 (100)
		Corynebacterium striatum	1	1 (100)
		Enterococcus casseliflavus	1	1 (100)
		Staphylococcus aureus	4	4 (100)
		Staphylococcus capitis	2	2 (100)
		Staphylococcus epidermidis	3	3 (100)
		Staphylococcus haemolyticus	5	5 (100)
		Staphylococcus hominis	3	3 (100)
		Staphylococcus sciuri	1	1 (100)

		Streptococcus agalactiae	3	3 (100)
		Streptococcus dysgalactiae	1	1 (100)
		Streptococcus mitis	1	1 (100)
		Streptococcus parasanguinis	1	1 (100)
	Fungi	*Candida parapsilosis*	1	1 (100)
Sputum	Gram-negative bacteria	*Acinetobacter baumannii*	12	12 (100)
(64)		*Acinetobacter lwoffii*	1	1 (100)
		Escherichia coli	3	3 (100)
		Klebsiella pneumoniae	17	17 (100)
		Morganella morganii	1	1 (100)
		Proteus mirabilis	2	2 (100)
		Pseudomonas aeruginosa	5	5 (100)
		Pseudomonas sp.	1	1 (100)
	Mycobacteria	*Mycobacterium* sp.	9	9 (100)
	Gram-positive bacteria	*Staphylococcus aureus*	3	3 (100)
		Staphylococcus epidermidis	1	1 (100)
		Staphylococcus haemolyticus	1	1 (100)
		Staphylococcus hominis	2	2 (100)
		Streptococcus agalactiae	1	1 (100)
		Streptococcus gallolyticus	1	1 (100)
		Streptococcus pneumoniae	1	1 (100)
		Streptococcus suis	1	1 (100)

Fungi		*Candida albicans*	1	1 (100)
		Candida tropicalis	1	1 (100)
Pus (5)	Gram-negative bacteria	*Escherichia coli*	2	2 (100)
		Enterobacter cloacae	1	1 (100)
	Gram-positive bacteria	*Staphylococcus aureus*	1	1 (100)
		Enterococcus faecalis	1	1 (100)
Urine (80)	Gram-negative bacteria	*Acinetobacter baumannii*	13	13 (100)
		Burkholderia cepacia	1	1 (100)
		Escherichia coli	17	17 (100)
		Klebsiella aerogenes	1	1 (100)
		Klebsiella pneumoniae	17	17 (100)
		Morganella morganii	1	1 (100)
		Pseudomonas aeruginosa	5	5 (100)
		Salmonella sp.	3	3 (100)
	Gram-positive bacteria	*Enterococcus faecalis*	3	3 (100)
		Staphylococcus aureus	8	9 (100)
		Staphylococcus epidermidis	1	1 (100)
		Staphylococcus haemolyticus	1	1 (100)
		Staphylococcus hominis	3	3 (100)
		Streptococcus agalactiae	1	1 (100)
		Streptococcus pyogenes	2	2 (100)
		Streptococcus sanguinis	1	1 (100)
		Streptococcus suis	2	2 (100)

Body fluid	Gram-negative bacteria	*Acinetobacter baumannii*	1	1 (100)
(9)		*Escherichia coli*	1	1 (100)
		Enterobacter cloacae	1	1 (100)
		Klebsiella pneumoniae	2	2 (100)
		Klebsiella aerogenes	1	1 (100)
	Gram-positive bacteria	*Staphylococcus aureus*	3	3 (100)
